# Four emerging immune cellular blood phenotypes associated with disease duration and activity established in Psoriatic Arthritis

**DOI:** 10.1186/s13075-022-02956-x

**Published:** 2022-11-29

**Authors:** Marie Skougaard, Sisse B. Ditlev, Zara R. Stisen, Laura C. Coates, Karen Ellegaard, Lars Erik Kristensen

**Affiliations:** 1 The Parker Institute, Bispebjerg and Frederiksberg Hospital, Frederiksberg, Denmark; 2 Department of Clinical Immunology, Aarhus University Hospital, Aarhus, Denmark; 3 Copenhagen Center for Translational Research, Bispebjerg and Frederiksberg Hospital, Copenhagen, Denmark; 4 Nuffield Department of Orthopaedics, Rheumatology and Musculoskeletal Sciences, University of Oxford, Oxford, UK

**Keywords:** Psoriatic Arthritis, Immunophenotyping, Flow cytometry, Principal Component Analysis, T cell

## Abstract

**Background:**

Psoriatic Arthritis (PsA) is an immune-mediated disease with heterogenous symptoms indicating differences in the underlying immunopathogenesis. The primary objective of the study explored the dynamic mechanisms and interplay between immune cell subtypes constituting the immune response driving PsA to evaluate possible differences in immune cellular phenotypes, and secondary examined associations between emerging immune cellular phenotypes and disease outcomes.

**Methods:**

Peripheral blood was collected from 70 PsA patients. Frequencies of nine immune cell subtypes were determined by multicolor flow cytometry. The interplay between immune cells were examined with principal component analysis (PCA) to establish immune cellular phenotypes. Disease characteristics, Disease Activity in Psoriatic Arthritis (DAPSA) and Psoriasis Area Severity Index (PASI) were retrieved to examine associations to individual cellular phenotypes.

**Results:**

Four components were identified using PCA resembling four immune cellular phenotypes. Component 1, explaining 25.6% of the variance with contribution from T-helper 17 cells (Th17), memory T regulatory cells (mTregs), dendritic cells and monocytes, was associated with longer disease duration and higher DAPSA. Component 2, driven by Th1, naïve Tregs and mTregs, was associated with shorter disease duration. Component 3 was driven by both Th1, Th17 and CD8+ T cells, while component 4 was characterized by a reverse correlation between CD8+ T cells and natural killer cells.

**Conclusion:**

Four immune cellular phenotypes of PsA were suggested at baseline demonstrating complex immune cellular mechanisms in PsA implying the possibility of improving PsA patient stratification based on both clinical and immune cellular phenotypes.

**Supplementary Information:**

The online version contains supplementary material available at 10.1186/s13075-022-02956-x.

## Background

Psoriatic Arthritis (PsA) is an immune-mediated disease with inflammation of involved tissues, including joints and entheses, causing swelling and tenderness [1]. Though diagnosed with the same disease, PsA patients suffer from diverse clinical manifestations that might include skin psoriasis, nail psoriasis and dactylitis [2]. This demonstrates the heterogeneity in clinical phenotypes observed when comparing PsA patients, and it further indicates the potential heterogeneity in the underlying immune cellular phenotypes. However, differences in immune cellular mechanisms differentiating PsA patients remain poorly understood.

PsA was originally believed to be driven by an autoimmune adaptive immune response [3], which has been challenged by evidence implicating the importance of an ongoing interplay between adaptive and innate mechanisms [4]. Innate immune cells, such as monocytes, are known to differentiate into antigen-presenting cells, including dendritic cells [5], found relevant to PsA due to their ability to secrete interleukin (IL)-12 and IL-23 inducing differentiation of T cells towards subtypes; type 1 and 17 helper T cells (Th1 and Th17) [1, 6]. T cells have been the primary suspects in PsA pathogenesis [7, 8], with IL-23 and IL-17 secreting CD4+ Th17 cells increased in both peripheral blood and synovial fluid [9]. Thus, both innate and adaptive immune cells have been shown to produce and release IL-17 [10] and an accumulation of IL-17 secreting cells has been found in joints of PsA patients and may further include IL-17 secreting CD8+ T cells [11]. T cell subsets, including both Th1, Th17 and CD8+ cytotoxic T (Tc) cells, are known mediators of inflammation in PsA leading to the notion of different immune cellular pathways in PsA [12]. Additionally, individualized treatment strategies targeting individual T cell phenotypes have been suggested [13]. Treatments that may alter immune cellular composition [14, 15], which is reportingly also seen to T-regulatory cells (Tregs) [15]. Tregs were originally considered to prevent chronic inflammation by inducing an anti-inflammatory effect [16]. However, studies on psoriasis and arthritis have revealed contradictory results regarding Treg levels in association with disease activity [17–19].

The dynamic mechanisms between individual immune cells driving PsA immunopathogenesis are complex and clarification is desired to improve patient stratification. Here we present results from an exploratory flow cytometry study assessing the frequencies of nine immune cell subtypes in 70 PsA patients. The primary objective was to assess the interplay between the nine immune cell subtypes to define immune cellular phenotypes in PsA patients, and secondary to explore the associations between emerging phenotypes and clinical outcome measures.

## Materials and methods

### Study population

Seventy PsA patients were recruited from the Parker Institute’s PsA patient cohort, which comprises an observational cohort study examining the effect of treatment on clinical, para-clinical and patient-reported parameters [20]. All patients participated in a baseline visit adjacent to initiating treatment. Patients were included if fulfilling the CASPAR criteria [19] and initiating new treatment with either Tumour Necrosis Factor alpha inhibitor (TNFi), Interleukin-17 inhibitor (IL-17i) or methotrexate (MTX).

The study was conducted in accordance with the Declaration of Helsinki with ethical approval obtained from the Danish Ethical Committee of the Capital Region of Denmark (J.no.: H-18024568), and the General Data Protection Regulation approved by the Capital Region of Denmark (J.no.: BFH-2015-043). All patients provided written informed consent before inclusion. The study was registered at clinicaltrials.gov (NCT02572700). A pre-specified protocol is publicly available at the Parker Institute’s website (www.parkerinst.dk).

### Patient involvement

A patient research partner was consulted to review research ideas and study objectives during the project. All results were presented and discussed in lay terms with the patient research partner.

### Variables and outcome measures

Clinical outcomes measures, i.e., 66/68 swollen and tender joint count, and patient-reported outcome, i.e., Visual Analogue Scale (VAS) patient global and pain, were obtained to retrieve the joint disease composite measure Disease Activity for Psoriatic Arthritis (DAPSA). Additional, clinical outcomes included evaluation of dactylitis (number), Spondyloarthritis Research Consortium of Canada (SPARCC) enthesitis, nail psoriasis (number), and Psoriasis Area Severity Index (PASI) was obtained to evaluate the psoriatic skin component.

### Preparation of cells

Peripheral blood was collected in EDTA vacutainer tubes (Greiner bio-one, Kremsmünster, Germany) and separated by erythrocyte lysis using Ammonium-Chloride-Potassium lysis (ACK) buffer (Lonza, Walkersville, MU USA) to obtain buffy coat. Buffy coat was cryopreserved in heat-inactivated fetal bovine serum (FBS) (Gibco, Grand Island, NY USA) with 10% dimethylsulphoxide (DMSO) (ITW Reagents, Darmstedt, Germany). Controlled freezing to -80 °C was ensured with CoolCell™ (Corning) cryogenic storage box and subsequently transferred to liquid nitrogen for longtime storage.

### Flow cytometry

Buffy coat was thawed and washed twice in RPMI (Gibco, Parsley, UK) with 10% FBS. Cell solution was diluted to aliquots of 1x10^6^ cells. Staining panels (Additional file 1) were adapted to local conditions from the Human Immunology Project [21]. The quantity of nine immune cell subtypes, defined by their cell surface markers, were evaluated; CD3+CD8+ Tc cells, CD3+CD4+CXCR3+CCR6- Th1 cells, CD3+CD4+CXCR3-CCR6+ Th17 cells, CD4+CD25+CD127^low^CD45RO- naïve Tregs (nTregs), CD4+CD25+ CD127^low^CD45RO+HLA-DR- Unactivated Memory Tregs (umTregs), CD4+ CD25+CD127^low^CD45RO+HLA-DR+ Activated Memory Tregs (amTreg), CD3-CD19-CD20-CD14-HLA-DR+ dendritic cells, CD3-CD19-CD20-CD14-CD16+ Natural killer cells (NK cells), and CD3-CD19-CD14+ Monocytes. Flow cytometry was conducted on the Beckman Coulter Gallios with three lasers and a 10-colours configuration. Gating strategy was established, and data was analyzed with Kaluza software for Gallios (Additional file 2).

### Statistical analysis

Patient characteristics were presented as medians with interquartile range (IQR) for continuous variables and numbers with percentages for categorical variables. To justify the comparison of cell populations from three staining panels, cell frequency was given as the percentage of the lymphocyte population, which were defined by forward/side scatter plot analyzed under the same circumstances. Principal component analysis (PCA) was incorporated for dimensionality reduction exploring the variance and correlation between evaluated immune cells at baseline. PCA is a well-established method for investigating phenotypes on both the clinical and molecular levels [22, 23]. The number of components included for interpretation was decided by the Kaiser criterion, including components with eigenvalues > 1.0 [24]. The contribution of individual immune cell subtypes to the component was considered important if being above the expected average contribution for all cell types (~11.1%). Component coefficients were evaluated for cell subtypes of importance. Component coefficients >0.50 were considered strong. Five ancillary PCAs were implemented as sensitivity analyses evaluating components when stratifying PsA patients by 1) type of treatment, i.e. TNFi, IL-17i and MTX and 2) clinical phenotype, i.e. PsA with concurrent psoriasis and PsA without psoriasis. Evaluating the secondary objective, patients were stratified by their median contribution to the component to evaluate differences in clinical outcomes between groups. Kruskal-Wallis test was applied to compare treatment groups, i.e., TNFi, IL-17i and MTX. Mann-Whitney U-test was included for the comparison of patient groups below and above the contribution median, respectively. Two-sided *p*-values <0.05 were considered statistically significant. Statistical analysis was carried out using *R Statistics* with additional package *factoextra.*

## Results

Seventy PsA patients initiating TNFi, IL-17i or MTX were included. No statistically significant differences were found at baseline comparing the three treatment groups with regards to age, sex, disease duration and clinical outcomes (Table 1). Statistically significant differences were found between groups comparing previous biological disease modifying anti-rheumatic drug (bDMARD) treatment and concomitant conventional synthetic DMARD (csDMARD).

**Table 1:** Patient characteristics

	All (***n***=70)	TNFi (***n***=30)	IL17 (***n***=20)	MTX (***n***=20)	***p***-value
Female	39 (55.71)	17 (56.67)	12 (60.00)	10 (50.00)	0.811
Age, years	51.50 (45.23-61.23)	49.25 (41.83-59.12)	50.15 (45.17-61.95)	56.80 (51.35-62.38)	0.133
Disease duration, years	4.00 (1.58-10.00)	3.67 (1.56-8.31)	7.75 (1.65-14.25)	3.13 (1.44-10.75)	0.645
**Treatment**					
Previous bDMARD					
Bio-naïve	40 (57.14)	20 (66.67)	2 (10.00)	18 (90.00)	< 0.001
1	15 (21.43)	7 (23.33)	6 (30.00)	2 (10.00)	
2	8 (11.43)	2 (6.67)	6 (30.00)	0 (0.00)	
3	3 (4.29)	0 (0.00)	3 (15.00)	0 (0.00)	
4	3 (4.29)	1 (3.33)	2 (10.00)	0 (0.00)	
5	1 (1.43)	0 (0.00)	1 (5.00)	0 (0.00)	
Concomitant csDMARD	22 (31.43)	13 (43.33)	9 (45.00)	0 (0.00)	0.016
Clinical phenotypes					
DAPSA	29.20 (21.80-39.90)	27.90 (21.85-39.73)	36.70 (26.00-47.45)	27.55 (20.50-36.83)	0.187
PASI	1.20 (0.00-3.30)	1.50 (0.30-3.40)	0.08 (0.00-2.83)	0.95 (0.00-2.93)	0.570
PsA without psoriasis	22 (31.43)	8 (26.67)	8 (40.00)	6 (30.00)	0.469
Dactylitis	16 (22.86)	6 (20.00)	6 (30.00)	4 (20.00)	0.869
Enthesitis	54 (77.14)	24 (80.00)	16 (80.00)	14 (70.00)	0.553
Nail psoriasis	43 (61.43)	19 (63.33)	10 (50.00)	14 (70.00)	0.383
**Immune cellular frequency**					
CD8+ T cells, %	19.98 (15.12-25.27)	21.02 (18.63-25.76)	18.51 (13.14-23.44)	17.70 (14.96-23.11)	0.086
Th1 cells, %	1.79 (1.28-2.96)	1.79 (1.50-3.43)	2.25 (1.48-2.94)	1.44 (1.05-2.47)	0.239
Th17 cells, %	3.32 (2.40-4.61)	3.25 (2.45-4.50)	3.73 (3.09-5.03)	2.81 (2.29-3.36)	0.089
nTregs, %	1.21 (0.87-1.48)	1.22 (0.98-1.56)	1.13 (0.78-1.50)	1.24 (0.82-1.44)	0.773
uamTregs, %	1.13 (0.89-1.50)	1.13 (0.95-1.42)	1.23 (0.86-1.62)	1.07 (0.79-1.56)	0.942
amTregs, %	0.38 (0.22-0.57)	0.36 (0.23-0.58)	0.51 (0.21-0.63)	0.36 (0.25-0.49)	0.760
Dendritic cells, %	1.21 (0.76-1.82)	1.30 (0.77-1.59)	1.18 (0.79-1.86)	1.10 (0.72-2.18)	0.900
NK cells, %	7.42 (5.04-9.69)	6.87 (5.16-9.18)	5.80 (4.00-8.69)	9.14 (6.07-11.41)	0.186
Monocytes, %	3.79 (2.08-5.41)	3.66 (2.03-4.85)	3.50 (2.11-5.13)	4.03 (3.41-8.13)	0.352

Patient demographics and treatment characteristics were presented as medians with corresponding interquartile range (IQR) for continuous variables and number of patients with corresponding percentage for categorical variables. Immune cellular frequencies were given as the median percentage of the lymphocyte population with corresponding IQR

*bDMARD* biological Disease Modifying Anti-rheumatic Drug, *csDMARD* conventional synthetic Disease Modifying Anti-Rheumatic Drug, *DAPSA* Disease Activity for Psoriatic Arthritis, *PASI* Psoriasis Area and Severity Index, *Th* T helper, *nTregs* naïve T regulatory cells, *uamTregs* unactivated memory T regulatory cells, *amTregs* Activated Memory Tregs, *NK cells* Natural Killer cells

### Four independent immune cellular phenotypes are defined in the PCA

Four components, explaining 66.5% of the variance in the dataset, were derived from the evaluation of eigenvalues. Component 1 contributed to 25.6% of the explained variance with Th17 cells, amTregs, umTregs, dendritic cells and monocytes as cell types of importance and main drivers of the component with contribution ≥11.1% and all with strong negative component coefficients (Table 2). Component 2, explaining 18.4% of the variance, was compared to Component 1 oppositely driven by Th1 cells, nTregs, umTregs and monocytes, thus with only Th1 cells and nTregs reaching a strong correlation (-0.65 and -0.73, respectively). Tc cells, Th1 cells and Th17 cells were considered important contributors to Component 3 that explained 12.5% of the variance. Only the Tc cells were strongly correlated to the component. Component 4 explained 10.0% of the total variance and was driven by CD8+ T cells and NK cells that were reversely correlated (0.61 and -0.59, respectively) (Table 2).

**Table 2:** Results from the Principal Component Analyses

	Contribution of individual cell type to the component (%)				Coefficients with correlation between cells type and components			
	Component				Component			
	1	2	3	4	1	2	3	4
Tc cells	2.35	1.32	**39.04**	**41.08**	-0.23	0.15	**-0.66**	**0.61**
Th1 cells	0.34	**25.46**	**19.09**	0.80	-0.09	**-0.65**	-0.46	-0.08
Th17 cells	**13.66**	0.23	**13.48**	7.21	**-0.56**	0.06	-0.39	-0.25
nTregs	0.07	**32.39**	0.15	0.01	0.04	**-0.73**	0.04	-0.01
amTregs	**25.31**	4.81	7.57	0.03	**-0.76**	-0.28	0.29	0.02
umTregs	**20.66**	**13.33**	5.55	1.18	**-0.69**	-0.47	0.24	0.10
Dendritic cells	**18.88**	1.79	0.64	0.08	**-0.66**	0.17	-0.08	0.03
NK cells	7.49	8.42	4.59	**39.46**	-0.42	0.37	-0.23	**-0.59**
Monocytes	**11.24**	**12.26**	9.89	10.14	**-0.51**	0.45	0.33	0.30

Important contribution to the component was defined as contribution above the average ~11.1%. Correlation coefficients >0.50 were considered strong. Bold text represent values of important contribution and strong correlation coefficients, respectively

*Tc* CD8+ cytotoxic T cells, *Th1* T helper cell type 1, *Th17* T helper cell type 17, *nTregs* naïve T regulatory cells, *amTregs* activated memory T regulatory cells, *umTregs* unactivated memory T regulatory cells, *NK cells* natural killer cells

### Associations between disease outcome measures and individual components

Comparing patients based on median contribution to the component demonstrated statistically significant differences between groups (Table 3). Assessing Component 1 revealed that PsA patients above-median contribution (0.708) had statistically significant higher DAPSA at baseline (median 37.20 [IQR; 25.80-42.10], *p*=0.004), longer disease duration (9.50 [1.92-15.71], *p*=0.014), and higher enthesitis score (median 5.00 [3.00-7.00], *p*<0.001) compared to patients with low contribution to the component (DAPSA 24.90 [19.15-36.40], disease duration 2.67 [1.92-5.21], enthesitis score (1.00 [0.00-3.00]). However, with statistically significant lower number of nails with psoriasis. Comparing patient groups associated with Component 2 demonstrated statistically significant difference in disease duration including patients with contribution to the component ≥median (0.713) having shorter disease duration (3.00 [1.00-8.13], *p*=0.039). No statistically significant differences were found comparing groups of Component 3, while statistically significant differences were found comparing PASI in Component 4 with lower PASI for the high contribution group (PASI 0.85 [0.00-1.58], *p*=0.046).

**Table 3:** Comparison of PsA patients stratified by median contribution to components of PCA

**Component 1**	≥ median contribution (0.708)	< median contribution (0.708)	*p*-value
Age	51.70 (45.40-60.10)	51.30 (45.00-62.80)	0.573
Disease duration, years	9.50 (1.92-15.71)	2.67 (1.92-5.21)	**0.004**
DAPSA	37.20 (25.80-42.10)	24.90 (19.15-36.40)	**0.014**
PASI	0.85 (0.00-3.60)	1.50 (0.40-3.15)	0.355
Dactylitis	0.00 (0.00-0.00)	0.00 (0.00-0.00)	0.948
Enthesitis	5.00 (3.00-7.00)	1.00 (0.00-3.00)	**<0.001**
Nail psoriasis	0.50 (0.00-6.00)	4.00 (1.00-8.00)	**0.025**
**Component 2**	≥ median contribution (0.713)	< median contribution (0.713)	*p*-value
Age	51.20 (40.80-60.55)	51.70 (45.40-61.50)	0.5809
Disease duration, years	3.00 (1.00-8.13)	5.83 (2.29-15.21)	**0.039**
DAPSA	32.30 (22.35-41.25)	28.90 (19.73-39.30)	0.418
PASI	1.05 (0.00-3.55)	1.20 (0.00-2.95)	0.990
Dactylitis	0.00 (0.00-1.00)	0.00 (0.00-0.00)	0.521
Enthesitis	3.00 (1.00-5.00)	3.00 (1.00-5.00)	0.749
Nail psoriasis	2.00 (0.00-5.00)	4.00 (0.00-8.00)	0.215
**Component 3**	≥ median contribution (0.655)	< median contribution (0.655)	*p*-value
Age	50.50 (42.40-59.20)	54.10 (46.40-62.10)	0.192
Disease duration, years	4.00 (2,13-10.00)	3.42 (1.00-12.13)	0.530
DAPSA	32.30 (23.45-39.70)	26.20 (20.93-41.15)	0.653
PASI	1.30 (0.00-3.23)	1.20 (0.00-3.00)	0.765
Dactylitis	0.00 (0.00-0.00)	0.00 (0.00-1.00)	0.569
Enthesitis	3.00 (0.00-5.00)	3.00 (1.00-6.00)	0.749
Nail psoriasis	3.00 (0.00-7.00)	3.00 (0.00-7.00)	0.978
**Component 4**	≥ median contribution (0.579)	< median contribution (0.579)	*p*-value
Age	51.70 (45.00-61.05)	51.30 (46.40-61.00)	0.846
Disease duration, years	3.42 (1.54-10.00)	4.00 (1.58-13.13)	0.703
DAPSA	31.05 (21.25-41.42)	29.00 (21.90-38.55)	0.824
PASI	0.85 (0.00-1.58)	2.00 (0.20-5.00)	**0.046**
Dactylitis	0.00 (0.00-0.00)	0.00 (0.00-0.00)	0.935
Enthesitis	3.00 (0.00-5.00)	3.00 (1.00-5.00)	0.767
Nail psoriasis	3.00 (1.00-8.00)	1.00 (0.00-5.00)	0.191

*PsA* Psoriatic Arthritis, *PCA* Principal Component Analysis, *DAPSA* Disease Activity for PSoriatic Arthritis, *PASI* Psoriasis Area Severity Index

### Similar immune cellular phenotypes emerged stratifying by treatment group

90% of PsA patients initiating MTX were bio-naïve, i.e., patients had not received any immunosuppressives with an assumed effect on the inflammatory immune response. Four components were included for analysis, explaining a total of 79.8% of the variance in the dataset comprising MTX initiators. In line with the primary analysis, the first component (29.3%) was driven by strongly correlated cells, Th17 cells (-0.66), amTregs (-0.88), umTregs (-0.76) and dendritic cells (-0.70), while the second component constituted 22.0% of the variance comprised correlated Tc cells (0.51), Th1 cells (0.80) and Th17 cells (0.52). Additionally, the fourth component was driven by Tc cells with contribution from umTregs, the last of which was not strongly correlated to Component 4 (Table 4). The results establish the importance of the proposed immune cellular phenotypes in PsA. Minor differences were seen evaluating principal components of PsA patients initiating immunosuppressives, i.e., TNFi and IL-17i initiators. Components of TNFi initiators implied shared contributions from multiple immune cell types, while principal components of IL-17i initiators resembled phenotypes of the primary analysis and MTX initiators (Additional files 3 and 4).

**Table 4:** Results from the Principal Component Analyses including MTX initiators

	Contribution of individual cell type to the component (%)				Coefficients with correlation between cells type and components			
	Component				Component			
	1	2	3	4	1	2	3	4
Tc cells	4.05	**13.03**	3.16	**47.33**	0.33	**0.51**	-0.22	**0.69**
Th1 cells	0.03	**32.06**	5.12	0.01	-0.03	**0.80**	0.28	0.01
Th17 cells	**16.44**	**13.53**	0.57	8.33	**-0.66**	**0.52**	0.09	-0.29
nTregs	4.04	10.89	**32.60**	0.31	-0.32	-0.46	**0.71**	0.06
amTregs	**29.69**	0.93	0.41	5.37	**-0.88**	-0.14	0.08	0.23
umTregs	**21.94**	1.93	2.11	**20.86**	**-0.76**	0.20	0.18	0.46
Dendritic cells	**18.77**	0.04	9.71	7.91	**-0.70**	0.03	-0.39	-0.28
NK cells	0.05	**26.00**	0.71	9.17	-0.04	**0.72**	0.01	-0.30
Monocytes	5.00	1.60	**46.33**	0.71	-0.36	-0.18	-0.18	0.08

Important contribution to the component was defined as contribution above the average ~11.1%. Correlation coefficients >0.50 were considered strong. Bold text represents values of important contribution and strong correlation coefficients, respectively

*MTX* methotrexate, *Tc* CD8+ cytotoxic T cells, *Th1* T helper cell type 1, *Th17* T helper cell type 17, *nTregs* naïve T regulatory cells, *amTregs* activated memory T regulatory cells, *umTregs* unactivated memory T regulatory cells, *NK cells* natural killer cells

### Deciphering PsA clinical heterogeneity by immune cellular phenotyping

Two PsA clinical phenotypes were defined as A) PsA patients with psoriasis (*n* = 48, PASI >0) and B) PsA patients without psoriasis (*n* = 22, PASI = 0). Four components were included for further analysis during the PCA (Additional files 5 and 6). Components explained a total of 67.8% and 81.4% of the variance in PsA patients with and without psoriasis, respectively. The two different clinical phenotypes (A and B) revealed similarities on the immune cellular level, with Component 1 constituting A) 27.2% and B) 31.0% of the variance defined by strong contribution from correlated cells Th17 (A: -0.59, B: -0.64), amTregs (A: -0.69, B: -0.85), umTregs (A: -0.67, B: -0.77), and dendritic cells (A: -0.54, B: -0.71). However, additional contribution was seen from strongly correlated monocytes to Component 1 in PsA patients with psoriasis (A: -0.55). Component 2 explained A) 16.6% and B) 25.4% of the variance with strong contribution from Th1 cells in both categories. However, Th1 cells did only exhibit strong correlation to other cells of Component 2 in PsA patients without psoriasis, i.e., Th1 (B: -0.76), nTregs (B: -0.82) and umTregs (B: -0.52), and not in PsA patients with psoriasis. Component 3 was driven by strongly correlated cells Tc (-0.53) and Th1 cells (-0.62) in PsA patients with psoriasis and oppositely correlated NK cells (0.57) and monocytes (-0.53) in PsA patients without psoriasis. In contrast, only NK cells (-0.51) and Tc (-0.74) contributed with strong correlation to Component 4 in PsA patients with and without psoriasis, respectively (Additional files 5 and 6).

## Discussion

The study aimed to assess immune cell characteristics evaluating possible differences in cellular phenotypes in PsA patients initiating new medical therapy. Comparing treatment groups, statistically significant differences were only found when comparing concomitant csDMARDs and number of previous bDMARDs. This, together with the observed difference in IL-17i initiators’ disease duration compared to TNFi and MTX initiators, may be explained by Danish treatment guidelines that include TNFi being first line bDMARD after csDMARD failure, while IL-17i would be considered after TNFi - unless TNFi was considered contraindicated.

The primary PCA demonstrated strong correlation between immune cell types resembling four immune cellular phenotypes in PsA. The four independent principal components of the PCA, i.e., four phenotypes were established demonstrating complex interplay between immune cells varying together might correspond to immune blood phenotypes in PsA. Patients with high contribution to Component 1, defined by the contribution of Th17, mTregs, dendritic cells and monocytes, had statistically significant longer disease duration and higher disease activity. Compared to patients contributing to Component 2, characterized by the contribution of Th1 and nTregs, who demonstrated significantly shorter disease duration, this may indicate two pathways including an early Th1 driven disease state and an established Th17 driven disease state with sustained inflammation and high disease activity in this patient cohort. This is supported by previous research reporting higher concentration of CXCL10, a chemokine known to recruit Th1 through CXCL3 [25] in PsA patients with early disease [26], and Th17 cells with reported strong correlation to disease activity together with their importance to both early and established PsA [27]. To fully understand the function of Tregs in this context, e.g. the association between nTregs to Th1 compared to the association between mTregs to Th17 in relation to patient characteristics, it is believed that further Treg subtyping is necessary. Component 3 resembles an immune cellular pathway of PsA including equal contribution of Th1 and Th17 previously described [13] though the strong correlation found with concomitant Tc cells is not well-defined. It may be hypothesized that IFNγ- and/or IL-17- producing Tc cells, found increased in PsA [28, 29], function in the correlating signaling pathway with Th1/Th17 to promote and sustain inflammatory arthritis. As for Tregs, further subtyping may be necessary in relation to the reverse relationship between Tc cells and NK cells observed in Component 4 that indicates the importance of Tc cells independent of Th1/Th17 influence in PsA. Overall, strong component coefficients demonstrate the strong correlation of individual immune cellular blood phenotypes in PsA. Different genotypes have been proposed to determine clinical phenotype [30] supporting the idea of differences in immune cellular phenotypes as well. However, it was not possible to demonstrate a clear distinction in immune cellular characteristics comparing clinical phenotypes, i.e., PsA patients with and without psoriasis. Though, results did show that Tc cells and monocytes were more significant contributors to components in PsA patients with psoriasis, whereas Th1 and NK cells seemed more important in PsA patients without psoriasis. However, larger studies including clinical homogeneous populations are needed to focus on and understand the immune cellular differences in different PsA clinical phenotypes. Due to the assumption that all immunosuppressive treatments would alter the immune response ancillary PCAs were performed stratifying by type of treatment. The findings supported the primary PCA by implying the importance of immune cell phenotypes comprised by 1) Th17 cells, mTregs and dendritic cells and 2) Tc, Th1, Th17 and NK cells in treatment naïve PsA patients, i.e. PsA patients with an ongoing inflammatory immune response that have not been modulated by medical therapies. Moreover, the ancillary PCAs demonstrated the importance of acknowledging that all treatments induce a yet poorly understood modulation of the inflammatory immune response. However, PCA including only IL-17i initiators that had experienced more therapy switching and suffered from continued high disease activity revealed immune cellular phenotypes resembling that of treatment naïve PsA patients.

The limitations of the study include that 90% of included IL-17i initiators had received other bDMARDs prior to initiation, which most likely have had an immune-modulatory effect compared to bio-naïve patients. Results are still considered of uttermost interest as these patients had high PsA disease activity with no difference to MTX and TNFi initiators. It may further be considered that immune-modulatory response to treatment may be dependent on the generic drug type used. In the current study 19 initiated adalimumab, 1 initiated certolizumab while 5 and 15 patients initiated secukinumab and ixekizumab, respectively, that have known different molecular properties causing diversity due to drug affinity [31, 32]. Moreover, immune cell frequencies were examined in peripheral blood which are known to differ, though, mirroring the immune milieu of inflammatory tissue [33]. As immune cellular milieu also differ comparing joint and skin [34], blood is considered to represent the immune milieu of both tissues. Moreover, additional cell subtyping of Tregs, T cells, monocytes etc. to address cell functionality including methods to evaluate cytokines or gene expression may add further important knowledge [35]. Still, it is believed that results from the current study provide valuable knowledge of immune cellular mechanisms in PsA and the different stages of disease.

## Conclusion

Immune cellular characteristics are complex and in-depth analysis of immune cell subtypes and interplay in PsA is needed to further understand the complex interdependence of immune cells in PsA immunopathogenesis. Results further add to the discussion of stratified treatment based on immune cellular phenotypes. However, additional understanding of immune cellular response mechanisms in larger cohorts is needed before treatment targeting individual immune cellular phenotypes will be possible.

## Supplementary Information

**Additional file 1.** Antibody panel for staining of cell surface markers

**Additional file 2.** Gating strategy

**Additional file 3.** Results from the Principal Component Analyses including TNFi initiators

**Additional file 4.** Results from the Principal Component Analyses including IL-17i initiators

**Additional file 5.** Results from the Principal Component Analyses in PsA patient with concurrent psoriasis

**Additional file 6.** Results from the Principal Component Analyses in PsA patient without psoriasis

## Data Availability

The data underlying this article cannot be shared publicly due to the privacy of the individuals that participated in the study. Data may be shared as part of research collaborations between participating institutions in line with GDPR and if approved by the Parker Institute and Danish authorities.

## Abbreviations

PsA: Psoriatic Arthritis
IL: Interleukin
Th1: T helper cell type 1
Th17: T helper cell type 17
Tc: Cytotoxic T cell
Tregs: T regulatory cells
TNFi: Tumour Necrosis Factor alpha inhibitor
IL-17i: Interleukin-17 inhibitor
MTX: Methotrexate
VAS: Visual Analogue Scale
DAPSA: Disease Activity in Psoriatic Arthritis
PASI: Psoriasis Area Severity Index
ACK: Ammonium-Chloride-Potassium lysis
FBS: Fetal bovine serum
nTregs: Naïve T regulatory cells
umTregs: Unactivated memory T regulatory cells
amTregs: Activated memory T regulatory cells
NK cells: Natural killer cells
IQR: Interquartile range
PCA: Principal components analysis
bDMARD: Biological disease modifying anti-rheumatic drug
csDMARD: Conventional synthetic disease modifying anti-rheumatic drug
