# Supplementary material for: Four emerging immune cellular blood phenotypes associated with disease duration and activity established in Psoriatic Arthritis
Source: Arthritis Res Ther. 2022 Nov 29;24:262. doi: 10.1186/s13075-022-02956-x (PMC9706839; doi:10.1186/s13075-022-02956-x)
Supplement: Supplementary file 1 — Additional file 1. Antibody panel for staining of cell surface markers [file 13075_2022_2956_MOESM1_ESM.docx]

|  | **Th1 / Th17** | **Manufac/ Cat.** | **Tregs** | **Manufac/ Cat.** | **Monocytes, DCs, NK cells** | **Manufac/ Cat.** |
| --- | --- | --- | --- | --- | --- | --- |
| PE | CXCR3 | BD/557185 | CD25 | BD/555432 | - | - |
| PerCP-Cy5.5 | CD4 | BD/560650 | CD4 | BD/560650 | - | - |
| PE-Cy7 | CCR6 | BD/560620 | - | - | - | - |
| APC | - | - | - | - | CD16 | BD/561304 |
| Alexa Flour 647 | - | - | CD127 | BD/558598 | - | - |
| APC-H7 | CD8 | BD/560179 | CD45RO | BD/560365 | CD3/CD19/CD20 | BD/560176  BD/560177  BD/560734 |
| V450 | CD3 | BD/560365 | CD3 | BD/560365 | CD14 | BD7560349 |
| V500 | - | - | HLA-DR | BD/561224 | HLA-DR | BD/561224 |

**Additional file 1** Antibody panel for staining of cell surface markers

Antibody panels were established based on the Human Immunology Project (21) and adapted to local conditions. PE; phycoerythrin, PerCP-Cy5.5; Peridinin-chlorophyl-protein cyanine 5.5, PE-Cy7; phycoerythrin cyanine 7, APC; allophycocyanin, APC-H7; allophycocyanin cyanine H7, V450; violet 450, V500; violet 500, CXCR; CXC-chemokine receptor, CD; cluster of differentiation, CCR; CC-chemokine receptor, HLA-DR; Human Leukocyte Antigen-DR, BD; Becton, Dickinson and Company
