# Supplementary material for: Four emerging immune cellular blood phenotypes associated with disease duration and activity established in Psoriatic Arthritis
Source: Arthritis Res Ther. 2022 Nov 29;24:262. doi: 10.1186/s13075-022-02956-x (PMC9706839; doi:10.1186/s13075-022-02956-x)
Supplement: Supplementary file 2 — Additional file 2. Gating strategy [file 13075_2022_2956_MOESM2_ESM.docx]

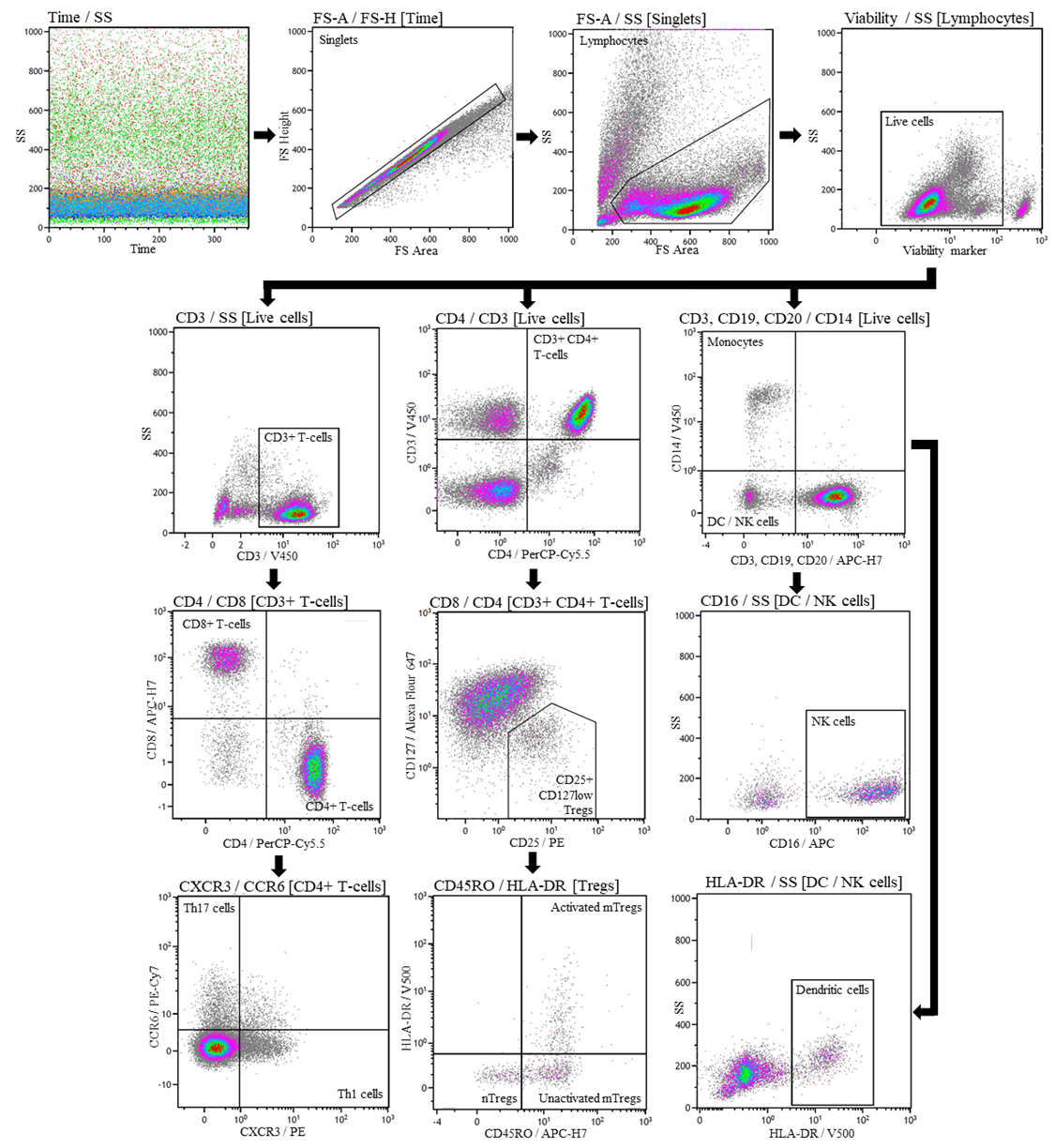
**Additional file 2:** Gating strategy

Gatring strategy to quantify 9 immune cell subsets. SS; side scatter, FS-A; forward scatter – area, FS-H; forward scatter – height, CD; cluster of differentiation, V450; violet 450, PerCP-Cy5.5; Peridinin-chlorophyl-protein cyanine 5.5, APC-H7; allophycocyanin cyanine H7, PE; phycoerythrin, APC; allophycocyanin, CXCR; CXC-chemokine receptor, PE-Cy7; phycoerythrin cyanine 7, Tregs; T regulatory cells, DC; dendritic cells, NK cells; natural killer cells, CCR; CC-chemokine receptor, HLA-DR; Human Leukocyte Antigen-DR, V500; violet 500, nTregs; naïve T regulatory cells, mTregs; memory T regulatory cells,
