# Supplementary material for: Four emerging immune cellular blood phenotypes associated with disease duration and activity established in Psoriatic Arthritis
Source: Arthritis Res Ther. 2022 Nov 29;24:262. doi: 10.1186/s13075-022-02956-x (PMC9706839; doi:10.1186/s13075-022-02956-x)
Supplement: Supplementary file 3 — Additional file 3. Results from the Principal Component Analyses including TNFi initiators [file 13075_2022_2956_MOESM3_ESM.docx]

|  | Contribution of individual cell type to the component (%) | | | | Coefficients with correlation between cells type and components | | | |
| --- | --- | --- | --- | --- | --- | --- | --- | --- |
|  | Component | | | | Component | | | |
|  | 1 | 2 | 3 | 4 | 1 | 2 | 3 | 4 |
| Tc cells | **11.29** | **16.09** | 0.47 | 9.09 | **0.50** | **-0.51** | 0.08 | -0.29 |
| Th1 cells | 2.43 | 1.91 | **24.67** | **39.52** | 0.23 | 0.18 | **0.60** | **0.60** |
| Th17 cells | 5.93 | **11.00** | **23.17** | **15.37** | 0.36 | -0.42 | **0.58** | -038 |
| nTregs | 1.24 | **10.18** | **35.80** | 6.46 | 0.16 | 0.40 | **0.72** | -0.24 |
| amTregs | **16.70** | **18.94** | 2.31 | 0.44 | **0.60** | **0.55** | -0.18 | -0.06 |
| umTregs | **14.74** | **24.38** | 0.53 | 0.22 | **0.57** | **0.63** | -0.09 | -0.04 |
| Dendritic cells | **17.64** | **14.60** | 0.76 | **15.49** | **0.62** | -0.48 | -0.11 | 0.38 |
| NK cells | **16.83** | 2.65 | 0.68 | 6.35 | **0.61** | -0.21 | -0.10 | 0.24 |
| Monocytes | **13.21** | **0.25** | **11.59** | 7.07 | **0.54** | 0.06 | -0.41 | -0.25 |

**Additional file 3:** Results from the Principal Component Analyses including TNFi initiators

Important contribution to the component was defined as contribution above the average ~11.1%. Correlation coefficients >0.50 were considered strong. Bold text represents values of important contribution and strong correlation coefficients, respectively. TNFi; Tumour Necrosis Factor alpha inhibitor, Tc; CD8+ cytotoxic T cells, Th1; T helper cell type 1, Th17; T helper cell type 17, nTregs; naïve T regulatory cells, amTregs; activated memory T regulatory cells, umTregs; unactivated memory T regulatory cells, NK cells; natural killer cells
