# Supplementary material for: Four emerging immune cellular blood phenotypes associated with disease duration and activity established in Psoriatic Arthritis
Source: Arthritis Res Ther. 2022 Nov 29;24:262. doi: 10.1186/s13075-022-02956-x (PMC9706839; doi:10.1186/s13075-022-02956-x)
Supplement: Supplementary file 4 — Additional file 4. Results from the Principal Component Analyses including IL-17i initiators [file 13075_2022_2956_MOESM4_ESM.docx]

|  | Contribution of individual cell type to the component (%) | | | | Coefficients with correlation between cells type and components | | | |
| --- | --- | --- | --- | --- | --- | --- | --- | --- |
|  | Component | | | | Component | | | |
|  | 1 | 2 | 3 | 4 | 1 | 2 | 3 | 4 |
| Tc cells | 1.82 | 0.52 | **63.14** | **-** | -0.23 | 0.11 | **-0.85** | **-** |
| Th1 cells | 7.47 | **23.57** | 0.56 | - | 0.46 | **0.71** | -0.08 | - |
| Th17 cells | **19.41** | 0.02 | 4.59 | - | **-0.75** | -0.02 | -0.23 | - |
| nTregs | 8.23 | **13.86** | 5.73 | - | 0.49 | **0.54** | -0.26 | - |
| amTregs | **14.01** | **18.91** | 1.57 | - | **-0.63** | **0.64** | 0.13 | - |
| umTregs | 5.24 | **29.14** | 3.11 | - | -0.39 | **0.79** | 0.19 | - |
| Dendritic cells | 10.43 | 5.84 | 7.86 | - | -0.55 | 0.35 | 0.30 | - |
| NK cells | **14.62** | 7.91 | 3.48 | **-** | **-0.65** | -0.41 | 0.20 | **-** |
| Monocytes | **18.78** | **0.23** | 9.95 | - | **-0.73** | -0.07 | -0.34 | - |

**Additional file 4:** Results from the Principal Component Analyses including IL-17i initiators

Important contribution to the component was defined as contribution above the average ~11.1%. Correlation coefficients >0.50 were considered strong. Bold text represents values of important contribution and strong correlation coefficients, respectively. IL-17i; Interleukin 17 inhibitor, Tc; CD8+ cytotoxic T cells, Th1; T helper cell type 1, Th17; T helper cell type 17, nTregs; naïve T regulatory cells, amTregs; activated memory T regulatory cells, umTregs; unactivated memory T regulatory cells, NK cells; natural killer cells
