# Supplementary material for: Four emerging immune cellular blood phenotypes associated with disease duration and activity established in Psoriatic Arthritis
Source: Arthritis Res Ther. 2022 Nov 29;24:262. doi: 10.1186/s13075-022-02956-x (PMC9706839; doi:10.1186/s13075-022-02956-x)
Supplement: Supplementary file 5 — Additional file 5. Results from the Principal Component Analyses in PsA patient with concurrent psoriasis [file 13075_2022_2956_MOESM5_ESM.docx]

**Additional file 5:** Results from the Principal Component Analyses in PsA patient with concurrent psoriasis

|  | Contribution of individual cell type to the component (%) | | | | Coefficients with correlation between cells type and components | | | |
| --- | --- | --- | --- | --- | --- | --- | --- | --- |
|  | Component | | | | Component | | | |
|  | 1 | 2 | 3 | 4 | 1 | 2 | 3 | 4 |
| Tc cells | 10.61 | 1.56 | **23.13** | 9.83 | -0.51 | 0.15 | **-0.53** | 0.30 |
| Th1 cells | 2.01 | **11.40** | **31.95** | **16.72** | -0.22 | -0.41 | **-0.62** | -0.39 |
| Th17 cells | **14.17** | 0.40 | **12.48** | **13.88** | -**0.59** | -0.08 | -0.39 | 0.36 |
| nTregs | 2.11 | **32.46** | 5.39 | 4.29 | -0.23 | **-0.70** | 0.26 | -0.20 |
| amTregs | **19.57** | 3.09 | 8.35 | 0.48 | **-0.69** | -0.21 | 0.32 | -0.07 |
| umTregs | **18.14** | **14.09** | 5.21 | 3.59 | **-0.67** | -0.46 | 0.25 | 0.18 |
| Dendritic cells | **11.85** | **13.64** | 1.30 | **16.37** | **-0.54** | 0.45 | -0.13 | -0.39 |
| NK cells | 9.03 | 9.41 | 1.19 | **27.87** | -0.47 | 0.37 | 0.12 | **-0.51** |
| Monocytes | **12.51** | **13.96** | **11.01** | 6.99 | **-0.55** | 0.46 | 0.37 | 0.26 |

**Table legend:** Important contribution to the component was defined as contribution above the average ~11.1%. Correlation coefficients >0.50 were considered strong. Bold text represent values of important contribution and strong correlation coefficients, respectively. Tc; CD8+ cytotoxic T cells, Th1; T helper cell type 1, Th17; T helper cell type 17, nTregs; naïve T regulatory cells, amTregs; activated memory T regulatory cells, umTregs; unactivated memory T regulatory cells, NK cells; natural killer cells
