# Supplementary material for: Four emerging immune cellular blood phenotypes associated with disease duration and activity established in Psoriatic Arthritis
Source: Arthritis Res Ther. 2022 Nov 29;24:262. doi: 10.1186/s13075-022-02956-x (PMC9706839; doi:10.1186/s13075-022-02956-x)
Supplement: Supplementary file 6 — Additional file 6. Results from the Principal Component Analyses in PsA patient without psoriasis [file 13075_2022_2956_MOESM6_ESM.docx]

**Table 2:** Results from the Principal Component Analyses in PsA patient without psoriasis

|  | Contribution of individual cell type to the component (%) | | | | Coefficients with correlation between cells type and components | | | |
| --- | --- | --- | --- | --- | --- | --- | --- | --- |
|  | Component | | | | Component | | | |
|  | 1 | 2 | 3 | 4 | 1 | 2 | 3 | 4 |
| Tc cells | 6.74 | 2.70 | 7.47 | **54.84** | 0.43 | 0.25 | -0.30 | **-0.74** |
| Th1 cells | 0.00 | **24.94** | 7.09 | 0.28 | 0.01 | **-0.76** | 0.30 | -0.05 |
| Th17 cells | **14.49** | 2.28 | **15.80** | 3.83 | **-0.64** | 0.23 | 0.44 | -0.20 |
| nTregs | 4.95 | **29.26** | 0.01 | 0.03 | 0.37 | **-0.82** | -0.01 | 0.02 |
| amTregs | **25.90** | 4.56 | 2.61 | 0.73 | **-0.85** | -0.32 | -0.18 | 0.09 |
| umTregs | **21.29** | **11.94** | 0.85 | 0.29 | **-0.77** | **-0.52** | -0.10 | -0.05 |
| Dendritic cells | **17.99** | 0.05 | **16.89** | **16.46** | **-0.71** | -0.03 | -0.46 | -0.41 |
| NK cells | 7.10 | **14.08** | **26.19** | 0.55 | -0.45 | **0.57** | **0.57** | -0.07 |
| Monocytes | 1.55 | 10.20 | **23.08** | **22.99** | -0.21 | 0.48 | **-0.53** | 0.48 |

**Table legend:** Important contribution to the component was defined as contribution above the average ~11.1%. Correlation coefficients >0.50 were considered strong. Bold text represent values of important contribution and strong correlation coefficients, respectively. Tc; CD8+ cytotoxic T cells, Th1; T helper cell type 1, Th17; T helper cell type 17, nTregs; naïve T regulatory cells, amTregs; activated memory T regulatory cells, umTregs; unactivated memory T regulatory cells, NK cells; natural killer cells
